# Psychometric validation of the SNAP-IV rating scale in amblyopic children at high AD/HD risk: structural validity and measurement invariance

**DOI:** 10.3389/fpsyt.2025.1655548

**Published:** 2025-10-28

**Authors:** Lu Pan, Meng Ru, Yuxing Huang, Wuqiang Luo, Lili Li, Yan Luo, Enwei Lin, Min Kong, Qi Chen, Yali Luo, Hairun Liu, Siyan Huang, Jin Zeng, Fei Han, Xin Xiao

**Affiliations:** ^1^ The School of Public Health, Guilin Medical University, Guilin, Guangxi, China; ^2^ Visual Science and Optometry Center, The People’s Hospital of Guangxi Zhuang Autonomous Region, Nanning, Guangxi, China; ^3^ School of Public Health, Guangxi Medical University, Nanning, Guangxi, China; ^4^ School of Public Health and Management, Guangxi University of Chinese Medicine, Nanning, Guangxi, China; ^5^ Cognitive Sleep Center, The People’s Hospital of Guangxi Zhuang Autonomous Region, Nanning, Guangxi, China; ^6^ Department of Ophthalmology, Guangdong Provincial People’s Hospital, Guangdong Academy of Medical Sciences, Southern Medical University, Guangzhou, China; ^7^ Guangxi Key Laboratory of Eye Health, The People’s Hospital of Guangxi Zhuang Autonomous Region, Nanning, Guangxi, China; ^8^ Guangxi Health Commission Key Laboratory of Ophthalmology and Related Systemic Diseases Artificial Intelligence Screening Technology, The People’s Hospital of Guangxi Zhuang Autonomous Region, Nanning, Guangxi, China; ^9^ Department of Scientific Research, The People’s Hospital of Guangxi Zhuang Autonomous Region, Nanning, Guangxi, China

**Keywords:** SNAP-IV, validation, reliability, amblyopia, AD/HD, children

## Abstract

**Objective:**

To validate the psychometric properties of the Swanson, Nolan, and Pelham Rating Scale (SNAP-IV) in amblyopic children at high AD/HD risk and establish its clinical utility for comorbid AD/HD screening.

**Methods:**

This cross-sectional study utilized baseline data from the China Amblyopia Behavioral Cohort (CABC), which comprises 465 amblyopic children (aged 4–17 years). The reliability of the SNAP-IV was comprehensively assessed using Cronbach’s alpha and the split-half coefficient. The validity of the SNAP-IV was evaluated using criterion validity with the Conners’ parent rating scale (CPRS) and construct validity via confirmatory factor analysis (CFA). The measurement invariance of the SNAP-IV across gender and age groups was also investigated.

**Results:**

The SNAP-IV demonstrated exceptional internal consistency (Cronbach’s α = 0.965 [95% CI: 0.958–0.972], split-half coefficient = 0.891) and strong criterion validity with the CPRS domains, particularly with respect to the oppositional factor of the SNAP-IV scale, which showed the highest correlation with the conduct problem factor of the Conners’ Parent Rating Scale (CPRS) (r_s_ = 0.837, 95% CI: 0.807–0.863, *p*<0.001, large effect). The findings indicated a substantial correlation between inattention and learning problems (r_s_ = 0.808, 95% CI: 0.767–0.834, *p*<0.001, large effect) and conduct problems (r_s_ = 0.719, 95% CI: 0.675–0.765, *p*<0.001, large effect). Confirmatory factor analysis confirmed a three-factor structure (inattention, hyperactivity/impulsivity, oppositional) with robust fit indices (*χ²*/(291) = 1033.4, χ²/df = 3.551, RMSEA=0.074, CFI=0.92, IFI=0.92), with full measurement invariance confirmed across gender and age groups.

**Conclusions:**

This study constitutes the first systematic validation of the SNAP-IV in amblyopic children, thereby establishing its robustness for AD/HD screening in visually impaired populations. The scale’s standardized application has the potential to enhance the screening of early AD/HD-amblyopia comorbidity and the development of multidisciplinary intervention strategies for integrating visual and behavioral rehabilitation.

## Introduction

Attention Deficit/Hyper Activity (AD/HD), a neurodevelopmental disorder characterized by inattention, hyperactivity, and impulsivity (1), has a global prevalence of approximately 4.8% in children (2), and an estimated prevalence of 9.8% in the United States (3, 4). Its persistence into adulthood (2) and profound impacts on cognitive, behavioral, and psychosocial functioning (5, 6) underscore the urgency of early identification and intervention. Emerging evidence reveals a notable comorbidity between amblyopia and AD/HD. The prevalence of amblyopia among children has been documented to be approximately 2.0% in South Korea (7),1.09% (95% CI: 0.86–1.35%) in central–southern China (8), and 0.99% in Taiwan, China (9). With respect to comorbidity risk, children diagnosed with amblyopia present a 68.7% increased risk of developing AD/HD (OR=1.687, 95% CI: 1.444–1.970) in the Republic of Korea (7), and a 1.81-fold elevated risk in Taiwan, China (HR=1.81, 95% CI: 1.59–2.06) (10). However, AD/HD patients present an 89% higher prevalence of amblyopia (OR=1.89, 95% CI: 1.76–2.05) (11). Given that amblyopia, a visual developmental disorder affecting 1.44% of preschoolers globally (12), shares pathophysiological mechanisms with AD/HD—such as striatal and subcortical neuronal dysfunction affecting executive attention and multisensory integration (13–16)—the co-occurrence of these disorders creates substantial clinical and economic burdens (17–19).

Despite these intersections, contemporary behavioral evaluations for AD/HD (e.g., the Conners’ Parent Rating Scale [CPRS] (20) and the Child Behavior Checklist [CBCL] (21)) lack specificity for populations affected by amblyopia (22). Furthermore, three significant limitations pertaining to the current validation of the SNAP-IV exist: (1) Methodological constraints: Previous studies have systematically excluded cohorts with visual impairments, notwithstanding the evidence that visual deficits can significantly modify behavioral phenotypes (e.g., irritability induced by visual fatigue) (23); (2) Cultural–theoretical deficiencies: The adaptation of the SNAP-IV for Chinese populations (24) remains unvalidated within sensory-impaired groups, prompting concerns regarding its generalizability; and (3) Clinical diagnostic complications: the attention dysfunction associated with amblyopia frequently overlaps with symptoms of visual impairment, resulting in diagnostic misattribution (17). These limitations pose a threat to timely identification and effective intervention strategies for AD/HD linked to amblyopia.

To address these gaps, this study conducted a systematic psychometric evaluation of the Chinese SNAP-IV in 465 amblyopic children (aged 4–17 years) from the China Amblyopia Behavioral Cohort (CABC). Utilizing a multidimensional validation framework, the present study examines internal consistency (Cronbach’s α), structural validity via confirmatory factor analysis (CFA), and measurement invariance across gender and age groups.

By establishing the reliability and validity of the SNAP-IV in this understudied population, our study aims to 1) provide evidence-based guidelines for integrating the SNAP-IV into amblyopia-associated AD/HD screening protocols, 2) increase diagnostic precision by clarifying behavioral manifestations related to visual impairment, and 3) reduce healthcare costs through early identification and multidisciplinary interventions. The present research addresses a critical methodological void (24–28) and offers a translational solution to improve behavioral health outcomes in visually impaired children.

## Methods

### Participants

This study recruited 465 amblyopic children aged 4–17 years from the China Amblyopia Behavioral Cohort (CABC), a multicenter initiative conducted between April 2024 and May 2026 across Guangxi, Guangdong, and Sichuan Provinces. The sample size was determined via an empirical approach (29), calculating 5–10 times the number of items in the SNAP-IV scale (26 items), yielding a minimum target of 130–260 participants.

The inclusion criteria for the study participants were as follows: The subject met the diagnostic criteria for amblyopia of the Chinese Medical Association Ophthalmology Branch (30), including anisometropic amblyopia (binocular spherical anisometropia ≥ 1.5D or cylindrical anisometropia ≥ 1.0D), ametropia amblyopia (binocular equivalent spherical anisometropia < 0.75D), and strabismic amblyopia; In addition, the subject had no other eye diseases or related systemic diseases; and no history of eye surgery. The exclusion criteria were as follows: The subject was found to be (1) unable to cooperate with the examination and (2) had other diseases that may affect the development of the central nervous system.

The severity classification criteria for amblyopia were as follows: (1) mild-to-moderate amblyopia, with best-corrected visual acuity (BCVA) lower than the age-specific normal value but ≤ 0.6 logMAR; and (2) severe amblyopia, with BCVA > 0.6 logMAR.

This study received ethical approval from the Ethics Committee of the Guangxi Zhuang Autonomous Region People’s Hospital (Approval No. KY-KJT-2023-285) and complied with the principles of the Declaration of Helsinki. Written informed consent was obtained from all participants and their guardians.

### Measures

#### Demographic data

The demographic data of the participants was collected via questionnaires, encompassing age, gender, parental education level, maternal smoking/alcohol history, family history of amblyopia, family history of myopia, and birth weight of amblyopic children, were collected through questionnaires.

#### Clinical data

The clinical data of the patients were collected through a comprehensive eye examination, including an assessment of visual acuity, a fundus examination, and an ocular alignment evaluation. At the initial visit, retinoscopy was performed using 1% atropine sulfate-induced ciliary muscle paralysis, and BCVA was converted to the logarithm of the minimum angle of resolution (LogMAR).

#### Behavioral questionnaires

The questionnaires were conducted through via the Questionnaire Star platform, with parents completing them under the guidance of professionals. The researchers then proceeded to review and process the collected questionnaires, identifying and addressing missing or anomalous values and removing invalid responses.

The SNAP-IV Rating Scale is comprised of 26 items (31, 32), which constitute three factors: inattention (9 items), hyperactivity/impulsivity (9 items), and oppositional (8 items). The 4-point Likert scale ranges from 0 (none) to 3 (extremely severe) (24) and is suitable for children aged 6–18 years. Although the SNAP-IV/CPRS are normed for children ages 6–18 years, prior adaptations have demonstrated adequate validity and reliability in younger children (aged 4–5 years) via parental reports (28, 33).

The conners parent rating scale (CPRS) used in the study is the 48-item version (34). The CPRS includes five subscales: conduct problem, learning problem, psychosomatic, hyperactivity/impulsivity, and anxiety. The scale utilized is a parallel 4-point rating scale, ranging from 0 to 3. This CPRS version was selected as the criterion measure for validating the SNAP-IV, primarily because of its extensive validation and establishment of normative data in Chinese populations, ensuring cultural appropriateness. The 48-item CPRS is a well-validated and optimal benchmark specifically for establishing criterion validity in this initial study, given its strong conceptual overlap with the SNAP-IV and proven utility in comparable Chinese research.

### Statistical analyzes

The data were analyzed via IBM SPSS Statistics 28.0 and AMOS 26.0. Normality was assessed via the Shapiro–Wilk test. Descriptive statistics (mean ± SDs) were used for normally distributed data; medians and interquartile ranges (IQR) were used for skewed data. Categorical variables are reported as frequencies/percentages (n, %). All the statistical tests were two-tailed, and a p-value < 0.05 was considered statistically significant. For comparisons involving multiple groups, Bonferroni-adjusted *post hoc* tests were applied to control for Type I error. To comprehensively convey the magnitude and precision of the findings, 95% confidence intervals (CIs) and specific effect sizes were reported for all key results. The specific effect size metrics used for each type of statistical test were listed as follow and were interpreted according to conventional thresholds.

#### Group comparisons

For demographic and clinical characteristics, Cramer’s V (phi (φ) coefficients or V) were used for chi-square tests, with interpretations as small (0.1), medium (0.3), or large (0.5); for non-normally distributed continuous variables, the effect size rank-biserial correlation (r_rβ_) was calculated alongside Mann-Whitney U tests, interpreted similarly to Cohen’s guidelines (small: 0.1, medium: 0.3, large: 0.5); and for parametric tests, Cohen’s d or η² were reported as appropriate.

#### Item analysis

Spearman’s rank correlation coefficients (r_s_) were calculated between items and factors, with the correlation coefficient (r_s_) itself interpreted as the effect size, reported with 95% confidence intervals (CI), where r values indicate effect size (small: 0.1-0.3, medium: 0.3-0.5, large: >0.5). Acceptable item–factor correlations were defined as those that were ≥ 0.40 (marginally acceptable) (35).

#### Reliability assessment

Cronbach’s α coefficient and split-half coefficients were used to evaluate the internal consistency of the scale and its factors. Which served as the primary reliability indices. A Cronbach’s α coefficient ≥ 0.70 was considered acceptable (36).

#### Validity evaluation

Criterion-related validity was analyzed via Spearman’s correlation analysis to examine the correlation between the SNAP-IV rating scale and each factor of the CPRS dimensions. The correlation coefficient (r_s_) and 95%CI was interpreted as the effect size. Structural validity was assessed via confirmatory factor analysis (CFA). The commonly used fit evaluation indicators are as follows: 1) χ²/df < 5.0 is an acceptable model; 2) Comparative Fit Index (CFI) and Incremental Fit Index (IFI) > 0.90 are acceptable standards; 3) Root Mean Square Error of Approximation (RMSEA) and Root Mean Square Residual (RMR) < 0.08 are acceptable standards. These fit indices serve as effect size measures for model adequacy. Convergent validity was supported by standardized factor loadings (λ) values > 0.50, composite reliability (CR) > 0.70 and average variance extracted (AVE) > 0.50 are ideal values (36–38). Discriminant validity was assessed using the Fornell-Larcker criterion by comparing the square root of the AVE for each factor with the inter-factor correlations. Additionally, the Maximum Shared Variance (MSV) and the Average Shared Variance (ASV) were calculated. Strong discriminant validity is indicated when the AVE for each construct is greater than its MSV, and simultaneously greater than its ASV.

#### Measurement invariance

Multigroup CFA was used to evaluate measurement invariance across gender and age. Invariance was tested by sequentially constraining the factor loadings (metric), intercepts (scalar), and residuals (strict) to be equal across groups. Invariance was supported if the change in fit indices between nested models met the following criteria: ΔCFI was < 0.01 and ΔRMSEA < 0.015 (39). These difference indices are the critical effect sizes for evaluating measurement invariance.

## Results

### Demographics and clinical characteristics of amblyopic children

A total of 465 amblyopic children (99.1% valid questionnaires) participated in this study, and their demographics and clinical features are summarized in **Table 1**. The sample had a mean age of 6.28 ± 1.87 years, with 67.96% of subjects being aged ≤ 6 years and 51.0% being boys. Amblyopia severity was predominantly mild-to-moderate (94.6%), with refractive amblyopia being the most prevalent subtype (63.6%). Stratified analyses (**Supplementary Table S1**) revealed no significant gender- or age-related differences in amblyopia severity (all *p* > 0.05, φ = 0.005–0.041, indicating negligible effects). However, severe amblyopia was associated with poorer BCVA in the worse eye (U=0, *p*<0.001, r_rβ_ = 0.398, medium effect) and was more prevalent in the anisometropic and strabismic subtypes (χ² = 17.2, *p*<0.001, Cramér’s V=0.192, small effect). Maternal smoking/alcohol history was low (< 5%), and 58.4% of the caregivers had a college-level education or higher. These results indicate that the sample was representative of typical clinical amblyopia populations.

**Table 1:** Demographic and clinical characteristics.

Characteristics	Total (N=465)
Gender (n (%))	
Boy	237 (51.0)
Girl	228 (49.0)
Age (years) (n (%))	
≤6	316 (67.96)
>6	149 (32.04)
Family history of amblyopia (n (%))	
Yes	30 (6.5)
No	435 (93.5)
Family history of myopia (n (%))	
Yes	87 (18.7)
No	378 (81.3)
Parental education (n (%))	
Middle school	103 (22.2)
High school	90 (19.4)
College or above	272 (58.4)
Mother’s smoking history (n (%))	
Yes	9 (1.94)
No	456 (98.06)
Mother’s history of alcohol consumption (n (%))	
Yes	38 (8.17)
No	427 (91.83)
Child’s birth weight (n (%))	
≤2500 g	69 (14.8)
2500~4000 g	376 (80.9)
≥4000 g	20 (4.3)
Types of amblyopia (n (%))	
Ametropic amblyopia	296 (63.6)
Anisometropic amblyopia	144 (31.0)
Strabismic amblyopia	25 (5.4)
Severity of amblyopia (n (%))	
Mild to moderate	440 (94.6)
Severe	25 (5.4)
BCVA (Median (P_25_, P_75_))	
Better eye	0.2 (0.1,0.3)
Worse eye	0.3 (0.2,0.4)
Subscales (Median (P_25_, P_75_))	
Inattention	0.67 (0.22,1.0)
Hyperactivity/Impulsivity	0.67 (0.22,1.0)
Oppositional	0.625 (0.125,1.0)

### Item analysis and factor correlations of the SNAP-IV

As shown in **Table 2**, all the factors were significantly positively correlated after Bonferroni correction (p < 0.001), with correlations ranging from moderate to strong (r_s_ = 0.724–0.923, large effect). The factor–total correlations (r_s_ = 0.887–0.923, large effect) were consistently greater than the correlations between factors (r_s_ = 0.724–0.768, large effect), supporting strong internal consistency. The strongest link was observed between inattention and hyperactivity/impulsivity (r_s_ = 0.768, 95% CI: 0.715–0.795, large effect), followed by moderate associations between inattention and oppositional behavior (r_s_ = 0.724, 95% CI: 0.677–0.766, large effect) and between hyperactivity/impulsivity and oppositional behavior (r_s_ = 0.739, 95% CI: 0.693–0.779, large effect). These results confirm the internal homogeneity of each factor and highlight meaningful relationships among core behavioral constructs.

**Table 2:** Spearman correlations of the interfactor and factor-total scores.

Subscales	Inattention	Hyperactivity/Impulsivity	Oppositional
Inattention	1.00		
Hyperactivity/Impulsivity	0.768 (0.715,0.795) ^***^	1.00	
Oppositional	0.724 (0.677,0.766) ^***^	0.739 (0.693,0.779) ^***^	1.00
Total	0.903 (0.885,0.919) ^***^	0.923 (0.908,0.936) ^***^	0.887 (0.865,0.905) ^***^

Values are Spearman’s correlation coefficients (r_s_) with 95% confidence intervals (95% CI) in parentheses.

*p < 0.05; **p < 0.01; ***p < 0.001.

### Internal consistency and reliability of the SNAP-IV

As demonstrated in **Table 3**, the reliability results for the SNAP-IV scale are presented. Cronbach’s α and split-half coefficients are retained as reliability metrics, which inherently reflect effect size-like properties for internal consistency. The Cronbach’s *α* for the total scale is 0.965 (95% CI: 0.958–0.972), and the Cronbach’s *α* values of each factor are greater than 0.90. The split-half reliability for the total scale is 0.891, and the individual factor coefficients range from 0.844–0.904. These metrics exceed the conventional psychometric thresholds (α ≥ 0.70), confirming that the SNAP-IV yields stable and reproducible scores in amblyopic children.

**Table 3:** Reliability of the SNAP-IV.

Subscales	Cronbach’s α	Split-Half reliability
Total	0.965	0.891
Inattention	0.929	0.904
Hyperactivity/Impulsivity	0.925	0.844
Oppositional	0.928	0.900

p<0.05*, p<0.01**, and p<0.001***.

### Criterion-related validity with the CPRS

The correlations with the CPRS revealed strong convergent validity (**Table 4**). Overall, the oppositional of the SNAP-IV exhibited the strongest associations, particularly with the conduct problem of the CPRS (r_s_ = 0.837, 95% CI: 0.807–0.863, *p*<0.001, large effect), indicating a close alignment between behavioral measures of oppositionality across the two instruments. Inattention was strongly correlated with both learning problems (r_s_ = 0.808, 95% CI: 0.767–0.834, *p*<0.001, large effect) and conduct problems (r_s_ = 0.719, 95% CI: 0.675–0.765, *p*<0.001, large effect), In contrast, correlations between the SNAP-IV subscales and the psychosomatic and anxiety factors of the CPRS were moderate (r_s_ = 0.346–0.478, p < 0.001, medium effects), indicating that these dimensions are less directly related to core ADHD symptom domains but still significantly overlap. Collectively, these results provide robust evidence that the SNAP-IV captures behavioral dimensions that are meaningfully associated with established CPRS constructs.

**Table 4:** Spearman correlations of the SNAP-IV and the CPRS.

Subscales	Inattention	Hyperactivity/Impulsivity	Oppositional
Conduct problem	0.719 (0.675,0.765) ^***^	0.710 (0.660,0.753) ^***^	0.837 (0.807,0.863) ^***^
Learning problem	0.808 (0.767,0.834) ^***^	0.676 (0.622,0.724) ^***^	0.677 (0.623,0.724) ^***^
Psychosomatic	0.475 (0.389,0.536) ^***^	0.423 (0.343,0.497) ^***^	0.478 (0.403,0.547) ^***^
Impulsivity/Hyperactivity	0.627 (0.567,0.681) ^***^	0.673 (0.618,0.721) ^***^	0.696 (0.644,0.741) ^***^
Anxiety	0.450 (0.371,0.520) ^***^	0.346 (0.261,0.426) ^***^	0.456(0.378,0.527) ^***^

Values are Spearman’s correlation coefficients (r_s_) with 95% confidence intervals (95% CI) in parentheses.

*p < 0.05; **p < 0.01; ***p < 0.001.

### Confirmatory factor analysis

**Figure 1:**
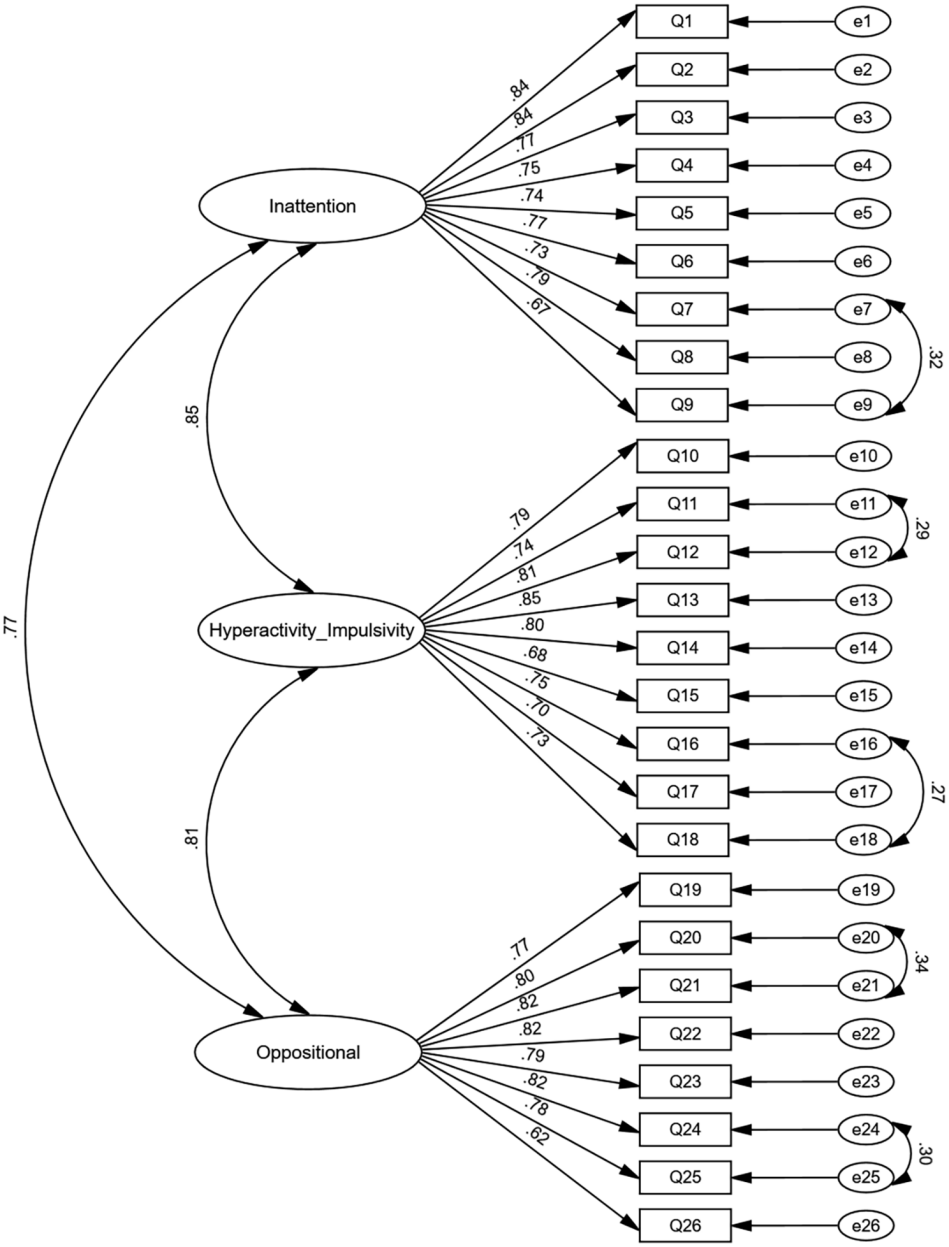
Standardized path coefficients of the SNAP-IV.

The initial three-factor model demonstrated suboptimal fit (*χ²*/(296) = 1225.6, *χ²*/df = 4.141, p < 0.05, RMSEA=0.082, CFI=0.90, IFI=0.90). Model refinement resulted in indices that met to acceptable levels (*χ²*/(291) = 1033.4, *χ²*/df = 3.551, *p*<0.05, RMSEA=0.074, CFI=0.92, IFI=0.92; **Figure 1**). The improved fit supports the scale’s structural validity in amblyopic populations.

### Convergent and discriminant validity

The results of the convergent validity study are presented in **Table 5**. Standardized factor loadings exceeded 0.5 (λ = 0.619–0.847, indicating large effects), with composite reliability (CR=0.924–0.928) and average variance explained (AVE=0.50–0.57) both meeting psychometric standards. The discriminant validity results in **Table 6** show that the correlation coefficients between the factors range from 0.767 to 0.851, with some exceeding the square root of their respective AVEs. The correlation coefficient between inattention and hyperactivity/impulsivity (*r* =0.851) is greater than the square root value of the AVE (0.769), and the correlation coefficient between hyperactivity/impulsivity and the oppositional factor (*r* =0.811) is greater than the square root value of the AVE (0.762), indicating that some factors are not independent enough and that the discriminant validity needs to be improved. Furthermore, we now include maximum shared variance (MSV=0.724) and average shared variance (ASV=0.657) as supplemental effect size metrics, confirming moderate-to-high shared variance but acceptable discriminant validity per Fornell-Larcker criterion. These indices collectively suggest that while the factors are robust, discriminant validity between them requires improvement.

**Table 5:** Standardized regression coefficients of the SNAP-IV.

Item			Estimate	S.E.	p	AVE	CR
Q1	<–––	Inattention	0.837			0.591	0.928
Q2	<–––	Inattention	0.841	0.044	***		
Q3	<–––	Inattention	0.772	0.046	***		
Q4	<–––	Inattention	0.754	0.041	***		
Q5	<–––	Inattention	0.740	0.042	***		
Q6	<–––	Inattention	0.771	0.044	***		
Q7	<–––	Inattention	0.731	0.048	***		
Q8	<–––	Inattention	0.790	0.049	***		
Q9	<–––	Inattention	0.667	0.045	***		
Q10	<–––	Hyperactivity/Impulsivity	0.794			0.581	0.925
Q11	<–––	Hyperactivity/Impulsivity	0.742	0.043	***		
Q12	<–––	Hyperactivity/Impulsivity	0.809	0.052	***		
Q13	<–––	Hyperactivity/Impulsivity	0.847	0.047	***		
Q14	<–––	Hyperactivity/Impulsivity	0.800	0.057	***		
Q15	<–––	Hyperactivity/Impulsivity	0.681	0.058	***		
Q16	<–––	Hyperactivity/Impulsivity	0.748	0.050	***		
Q17	<–––	Hyperactivity/Impulsivity	0.698	0.040	***		
Q18	<–––	Hyperactivity/Impulsivity	0.725	0.047	***		
Q19	<–––	Oppositional	0.772			0.605	0.924
Q20	<–––	Oppositional	0.804	0.055	***		
Q21	<–––	Oppositional	0.820	0.052	***		
Q22	<–––	Oppositional	0.793	0.052	***		
Q23	<–––	Oppositional	0.793	0.051	***		
Q24	<–––	Oppositional	0.822	0.054	***		
Q25	<–––	Oppositional	0.782	0.050	***		
Q26	<–––	Oppositional	0.619	0.040	***		

p<0.05*, p<0.01**, and p<0.001***.

**Table 6:** Discriminant validity of the SNAP-IV.

Variables	Inattention	Hyperactivity/Impulsivity	Oppositional
Inattention	0.591		
Hyperactivity/Impulsivity	0.851^***^	0.581	
Oppositional	0.767^***^	0.811^***^	0.605
Square root of AVE	0.769	0.762	0.778
MSV (Maximum Shared Variance)	0.724	0.724	0.724
ASV (Average Shared Variance)	0.657	0.657	0.657

Square root of AVE is compared with inter-factor correlations for discriminant validity. MSV and ASV are included as supplementary indicators of discriminant validity; p<0.05*, p<0.01**, and p<0.001***.

### Measurement invariance

Measurement invariance for gender and age groups was evaluated via the three-factor model, with the results summarized in **Table 7**. ΔCFI and ΔRMSEA are reported as effect sizes for model differences (e.g., for gender scalar invariance: ΔCFI=-0.002, small effect; ΔRMSEA=-0.001, negligible effect). Both groups showed an acceptable fit in the configural model (RMSEA=0.063, CFI=0.885, RMR=0.028 for gender; RMSEA=0.063, CFI=0.886, RMR=0.331 for age). The fit of the metric, scalar, and strict factor models was similar to that of the configural model (ΔRMSEA < 0.015, ΔCFI < 0.01), thereby supporting measurement invariance across gender and age groups.

**Table 7:** Multigroup CFA fit indices for the SNAP-IV across gender and age.

Model (gender)	χ2	df	Δχ2	Δdf	*p*	CFI	RMSEA	RMR	ΔCFI	ΔRMSEA
Model1 (configural invariance)	1669.454	592				0.885	0.063	0.028		
Model2 (metric invariance)	1690.035	615	20.581	23	0.607	0.885	0.061	0.031	0	-0.002
Model3 (scalar invariance)	1713.67	621	44.216	29	0.035	0.883	0.062	0.043	-0.002	-0.001
Model4 (strict invariance)	1770.387	647	100.933	55	0.000	0.88	0.061	0.044	-0.005	-0.002
Model (age)										
Model1 (configural invariance)	1664.644	592				0.886	0.063	0.031		
Model2 (metric invariance)	1691.426	615	26.782	23	0.265	0.886	0.061	0.037	0	-0.002
Model3 (scalar invariance)	1697.805	621	33.161	29	0.271	0.886	0.061	0.055	0	-0.002
Model4 (strict invariance)	1780.282	647	115.638	55	0.000	0.88	0.062	0.056	-0.006	-0.001

p<0.05*, p<0.01**, and p<0.001***.

## Discussions

### Key findings and psychometric robustness

This study represents the first systematic psychometric evaluation of the SNAP-IV rating scale in amblyopic children at high AD/HD risk, demonstrating exceptional reliability and validity. The scale exhibited outstanding internal consistency, surpassing previous cross-cultural adaptations in Brazil (*α* > 0.91) and China (*α* =0.88–0.90) (24, 28). These findings serve to reinforce the stability of the SNAP-IV in capturing homogenous behavioral constructs across diverse populations. The findings of the factorial analysis provided further support for the construct validity of the scale, with strong correlations between factors, indicating that all items reliably discriminate their respective dimensions (40, 41).

Importantly, the observed effect sizes across the main psychometric indices ranged from medium to large, highlighting that the associations observed are not only statistically significant but also of practical and clinical importance. Convergent validity with the CPRS was robust, particularly with respect to the oppositional factor of the SNAP-IV scale, which showed a large effect size correlation with the conduct problem factor of the CPRS (r_s_ = 0.837, *p <*0.001), which is consistent with evidence that children with unilateral visual impairment frequently present with heightened behavioral difficulties, including externalizing symptoms (23). Notably, large correlations between inattention and behavior problems (with learning problems and conduct problems, all *p <*0.001) underscore that attentional deficits exert a substantial behavioral impact in visually impaired children. Such magnitudes suggest that these relationships reflect meaningful behavioral mechanisms rather than trivial statistical findings. The large effect sizes observed support the hypothesis that attentional resource allocation deficits secondary to visual impairment, manifesting as cascading behavioral sequelae (42), thereby supporting theories of symptom overlap in neurodevelopmental disorders (43, 44). Together, these results demonstrate both statistical robustness and real-world relevance, underscoring the need for integrated interventions that addressing both visual symptoms and behavioral comorbidities in children with amblyopia.

### Factor structure and measurement invariance

The three-factor model of the SNAP-IV (inattention, hyperactivity/impulsivity, oppositional) is illustrated in **Figure 1**. This model demonstrated strong cross-cultural adaptability with acceptable fit indices (p < 0.05) (28, 41, 45). However, initial model misfit (RMSEA=0.082) highlighted insufficient factor independence, a finding that is probably attributable to the clinical co-occurrence of AD/HD symptoms (44) and nonspecific behavioral responses to visual dysfunction (46). As depicted in **Figure 1**, the standardized path coefficients ranged from 0.62 to 0.85, corresponding to large effect sizes, which affirm both the statistical robustness and the practical interpretability of the model. These magnitudes indicate that items have substantial loading strength on their latent constructs, advancing SNAP-IV understanding by confirming its factor structure in amblyopic children-a previously untested domain-and supporting its use for nuanced ADHD subtyping in visual impairment contexts. A high proportion of individuals ≤6 years of age (67.96%) may attenuate age norms, but invariance tests confirmed their applicability. Similarly, the measurement invariance across gender groups further corroborated the scale’s applicability across demographic subgroups.

However, configural and metric invariance were maintained (Δχ² = 26.782, Δdf = 23, *p* =0.265), and the small-to-medium effect size differences observed at the intercept level (ΔRMSEA = 0.001, ΔCFI = 0.006), which may reflect variations in behavioral expression due to age and environmental factors. This aligns with developmental trends showing declining hyperactivity with increasing age (20, 24). These effects are modest yet informative, emphasizing the need for age-adjusted thresholds in clinical practice. Furthermore, small effect size differences were detected across gender in scalar and strict invariance models (both *p*<0.05, ΔRMSEA < 0.005, ΔCFI < 0.002), with behavioral patterns consistent with clinical features of AD/HD (47). The minimal magnitude of these differences suggests they represent true behavioral variation rather than measurement bias. The small magnitude of these differences indicates they reflect genuine behavioral variation rather than measurement bias. Cross-cultural evidence suggests that these gender differences cannot be attributed to reporter bias or differences in assessment tools (48). These insights underscore the necessity of gender-sensitive assessment protocols in pediatric populations.

### Clinical and theoretical implications

The SNAP-IV scale has demonstrated strong reliability and validity across various cultural adaptations (28, 49, 50), underscoring its clinical value in assessing behavioral issues in amblyopic children. The validated reliability and validity of the SNAP-IV in this understudied population provide a critical tool for the early identification of AD/HD comorbidities in amblyopic children. Given that a high percentage of amblyopic children develop AD/HD-related behaviors (51) and that visual treatment windows is typically limited to age 7 (52), this scale offers timely clinical utility. Our findings align with transactional developmental theory (42, 53), suggesting that visual impairment disrupts attentional processes, which in turn exacerbate behavioral outcomes. The overlap between attentional difficulties and visual impairment may further complicate diagnostic processes, underscoring the need for precise, population-specific assessment protocols. This mechanistic framework justifies multidisciplinary interventions targeting both sensory and behavioral domains in children with amblyopia. From the perspective of multidisciplinary management of amblyopia, the results of this study provide a practical tool for the comprehensive assessment and intervention of children with amblyopia, ultimately leading to better long-term outcomes for these children.

### Limitations and future directions

While groundbreaking, this study has several limitations. First, parent-reported data introduced potential reporter bias, as both the SNAP-IV and the CPRS were completed by caregivers (54). Second, the absence of a clinical AD/HD diagnosis group precluded sensitivity/specificity analysis. Third, the cross-sectional design provided limited insights into the temporal stability of the scale. Fourth, both the authors and the participants in this study belong to the same ethnic and cultural group, potentially influencing the results through in-group familiarity with subtle behavioral norms (42). Caution is warranted when generalizing findings to other populations. Last but still important, while the SNAP-IV/CPRS lack age/gender norms (unlike Conners-4), they were chosen for validated Chinese adaptations in pediatric cohorts and brevity for clinical feasibility. Future research should incorporate teacher and self-reports to triangulate data, longitudinal tracking to assess developmental trajectories (55, 56), and validation against gold-standard diagnostic interviews (e.g., Conners-4) (57). Additionally, optimizing items for age-specific expression (e.g., preschool vs. school-age) and incorporating neurocognitive measures (e.g., eye-tracking, fMRI) could enhance discriminative validity (58).

## Conclusions

This study establishes the SNAP-IV as a reliable and valid instrument for assessing AD/HD-related behaviors in amblyopic children, filling a critical gap in developmental psychology research. While measurement invariance was largely preserved, age- and gender-related variations necessitate refined calibration for clinical use. Future advancements should prioritize longitudinal validation and multidisciplinary integration to address the complex interplay between visual impairment and behavioral comorbidities, ultimately improving long-term developmental outcomes for these vulnerable children.

## Data Availability

The raw data supporting the conclusions of this article will be made available by the authors, without undue reservation.
