# Supplementary material for: Psychometric validation of the SNAP-IV rating scale in amblyopic children at high AD/HD risk: structural validity and measurement invariance
Source: Front Psychiatry. 2025 Oct 28;16:1655548. doi: 10.3389/fpsyt.2025.1655548 (PMC12602427; doi:10.3389/fpsyt.2025.1655548)
Supplement: Supplementary file 1 [file Table1.docx]

| Characteristics | Age group | | | Gender group | | | Severity of amblyopia groups | | |
| --- | --- | --- | --- | --- | --- | --- | --- | --- | --- |
|  | ≤6(n=316) | >6(n=149) | *p* | Male(n=237) | Female(n=228) | *p* | Mild to moderate(n=440) | Severe(n=25) | *p* |
| Gender(n (%)) |  |  |  |  |  |  |  |  |  |
| Male | 163 | 74 | χ² = 0.15, p = 0.699 (φ = 0.019) | - | - | - | 224 | 13 | χ² = 0.011, p = 0.915 (φ = 0.005) |
| Female | 153 | 75 |  | - | - |  | 216 | 12 |  |
| Age (years) (n (%)) |  |  |  |  |  |  |  |  |  |
| ≤6 | - | - | - | 163 | 153 | χ² = 0.15, p = 0.699 (φ = 0.019) | 301 | 15 | χ² = 0.768, p = 0.381 (φ = 0.041) |
| >6 | - | - |  | 74 | 75 |  | 139 | 10 |  |
| Family history of amblyopia (n (%)) |  |  |  |  |  |  |  |  |  |
| Yes | 21 | 9 | χ² = 0.061, p = 0.804 (φ = 0.012) | 14 | 16 | χ² = 0.237, p = 0.626 (φ = 0.023) | 28 | 2 | χ² = 0.101, p = 0.754 (φ = 0.015) |
| No | 295 | 140 |  | 223 | 212 |  | 412 | 23 |  |
| Family history of myopia (n (%)) |  |  |  |  |  |  |  |  |  |
| Yes | 62 | 25 | χ² = 0.538, p = 0.463 (φ = 0.034) | 44 | 23 | χ² = 0.007, p = 0.935 (φ = 0.004) | 85 | 2 | χ² = 2.42, p = 0.121 (φ = 0.072) |
| No | 254 | 124 |  | 193 | 185 |  | 355 | 23 |  |
| Parental education (n (%)) |  |  |  |  |  |  |  |  |  |
| Middle school | 51 | 52 | χ² = 23.884, p < 0.001 (V = 0.217) | 51 | 52 | χ² = 0.203, p = 0.903 (V = 0.021) | 95 | 8 | χ² = 5.625, p = 0.064 (V = 0.110) |
| High school | 59 | 31 |  | 45 | 45 |  | 82 | 8 |  |
| College or above | 206 | 66 |  | 141 | 131 |  | 263 | 9 |  |
| Mother's smoking history (n (%)) |  |  |  |  |  |  |  |  |  |
| Yes | 3 | 6 | Fisher's p = 0.025 (φ = 0.093) | 5 | 4 | Fisher's p = 0.781 (φ = 0.019) | 9 | 0 | Fisher's p = 0.471 (φ = 0.032) |
| No | 313 | 143 |  | 232 | 224 |  | 431 | 25 |  |
| Mother's history of alcohol consumption (n (%)) |  |  |  |  |  |  |  |  |  |
| Yes | 24 | 14 | χ² = 0.438, p = 0.508 (φ = 0.031) | 18 | 20 | χ² = 0.215, p = 0.643 (φ = 0.022) | 35 | 3 | χ² = 0.463, p = 0.498 (φ = 0.032) |
| No | 292 | 135 |  | 219 | 208 |  | 405 | 22 |  |
| Child's birth weight (n (%)) |  |  |  |  |  |  |  |  |  |
| ≤2500 g | 46 | 23 | χ² = 1.743, p = 0.418 (V = 0.061) | 27 | 42 | χ² = 5.751, p = 0.056 (V = 0.111) | 65 | 4 | χ² = 0.005, p = 0.984 (V = 0.003) |
| 2500~4000 g | 259 | 117 |  | 197 | 179 |  | 356 | 20 |  |
| ≥4000 g | 11 | 9 |  | 13 | 7 |  | 19 | 1 |  |
| Types of amblyopia (n (%)) |  |  |  |  |  |  |  |  |  |
| Ametropic amblyopia | 209 | 87 | χ² = 2.635, p = 0.268 (V = 0.075) | 149 | 147 | χ² = 0.129, p = 0.937 (V = 0.017) | 291 | 5 | χ² = 17.2, p < 0.001 (V = 0.192) |
| Anisometropic amblyopia | 91 | 53 |  | 75 | 69 |  | 128 | 16 |  |
| Strabismic amblyopia | 16 | 9 |  | 13 | 12 |  | 21 | 4 |  |
| Severity of amblyopia (n (%)) |  |  |  |  |  |  |  |  |  |
| Mild to moderate | 301 | 139 | χ² = 0.768, p = 0.381 (φ = 0.041) | 224 | 216 | χ² = 0.011, p = 0.915 (φ = 0.005) | - | - | - |
| Severe | 15 | 10 |  | 13 | 12 |  | - | - |  |
| BCVA (Median (****IQR****)) |  |  |  |  |  |  |  |  |  |
| Better eye | 0.2(0.1,0.3) | 0.1(0,0.2) | U = 31574.5, p < 0.001 (r = 0.281) | 0.2（0.1,0.3） | 0.2（0.1,0.3） | U = 27916, p = 0.528 (r = 0.029) | 0.2(0.1,0.3) | 0.1(0,0.4) | U = 6218, p = 0.263 (r = 0.052) |
| Worse eye | 0.3(0.2,0.5) | 0.3(0.2,0.4） | U = 26467.5, p = 0.027 (r = 0.102) | 0.3（0.2,0.45） | 0.3（0.2,0.4） | U = 28507.5, p = 0.295 (r = 0.049) | 0.3(0.2,0.4) | 0.8(0.8,0.9) | U = 0, p < 0.001 (r = 0.398) |
| Subscales (Median (****IQR****)) |  |  |  |  |  |  |  |  |  |
| Inattention | 0.56(0.22,0.97) | 0.67（0.33,1.06） | U = 20148, p = 0.012 (r = 0.117) | 0.67（0.22,1.0） | 0.56（0.14,1.0） | U = 28739.5, p = 0.233 (r = 0.055) | 0.67(0.22,1.0) | 0.78(0.17,1.110 | U = 5050, p = 0.489 (r = 0.032) |
| Hyperactivity/Impulsivity | 0.67(0.22,1.0) | 0.67（0.1,1.0） | U = 24070.5, p = 0.695 (r = 0.018) | 0.78（0.33,1.11） | 0.56（0.11,1.0） | U = 32761.5, p < 0.001 (r = 0.185) | 0.67(0.22,1.0) | 0.67（0.33,1.11） | U = 4998, p = 0.575 (r = 0.036) |
| Oppositional | 0.625(0.125,1.0) | 0.75（0.375,1.0） | U = 21518.5, p = 0.132 (r = 0.070) | 0.75（0.25,1.0） | 0.625（0.125,1.0） | U = 29651.5, p = 0.067 (r = 0.085) | 0.625(0.125,1.0) | 0.75（0.19,1.0） | U = 5136, p = 0.441 (r = 0.026) |
